# Factors associated with the evaluation of geriatric assessment (GA) domains by oncology specialists in Mexico

**DOI:** 10.3332/ecancer.2023.1597

**Published:** 2023-08-31

**Authors:** Haydeé Cristina Verduzco-Aguirre, Laura Margarita Bolaño-Guerra, Javier Monroy Chargoy, Eva Culakova, Héctor Martinez-Said, Gregorio Quintero Beulo, Supriya G Mohile, Enrique Soto-Perez-de-Celis

**Affiliations:** 1 Instituto Nacional de Ciencias Médicas y Nutrición Salvador Zubirán, Mexico City 14080, Mexico; 2 Clínica Portoazul Auna, Barranquilla 081007, Colombia; 3 University of Rochester Medical Center, Rochester, NY 14642, USA; 4 Instituto Nacional De Cancerología, Mexico City 14080, Mexico; 5 Hospital General de México “Dr. Eduardo Liceaga,” Mexico City 06720, Mexico

**Keywords:** geriatric oncology, geriatric assessment, Mexico

## Abstract

The use of geriatric assessment (GA) by oncology specialists in Mexico is low. We aimed to explore factors associated with the evaluation of individual GA domains by Mexican oncology specialists. We performed an exploratory analysis of a sequential explanatory mixed-methods study consisting of an online cross-sectional survey of Mexican oncology specialists and follow-up interviews on the use of GA in cancer care. For each GA domain, we performed multivariable logistic regression analyses with the frequency of evaluation of the domains as the dependent variable (dichotomised as never/rarely/sometimes versus most of the time/always). A *p*-value <0.05 was considered significant. Qualitative data from the interviews were analysed inductively. Of 196 respondents, 62% were male, 50% were surgical oncologists, 51% took care of >10 patients per day and 61.7% had access to a geriatrician. Self-perceived confidence in managing common geriatric conditions was associated with the evaluation of specific GA domains. For instance, self-perceived confidence in managing dementia (OR 2.72; 95% CI 1.42–5.51, *p* = 0.008) was associated with cognition evaluation, while for evaluation of falls, self-perceived confidence in evaluation of falls (OR 6.31; 95% CI 3.19–12.46, *p* < 0.001) was significantly associated. Follow-up interviews showed quality and appropriateness of evaluations may not be ideal: in many cases, physicians do not use guideline-recommended tools. For example, evaluation of cognition is commonly performed through non-validated methods which may miss the detection of patients with an impairment in this domain, partly due to limitations in knowledge and time to use recommended tools. In conclusion, self-perceived confidence in evaluating and managing common situations in older adults was associated with the evaluation of GA domains as part of everyday practice in a sample of oncology specialists in Mexico. This analysis supports the use of educational interventions to boost knowledge and confidence regarding the proper use of validated GA tools among oncology specialists.

## Background

Medical care for older adults with cancer represents a high priority because of the rapid increase in global life expectancy, which has doubled in the last century [1]. Currently, over 50% of patients newly diagnosed with cancer are aged ≥65 years [2], and this number is expected to increase as the world population ages. In Latin America and the Caribbean, 48% of new cancer diagnoses occur in older individuals, and incidence will double by 2040 [3]. In Mexico, the number of individuals aged ≥65 is expected to increase by 8.5 million by 2030; thus, it is expected that 60% of cancer cases will present in older patients.

There is no universal cut-off point to define a person as an older adult, which reflects the fact that chronological age by itself is not enough to explain aging. However, for practical purposes, chronological age is used for said definition. In the field of oncology, and particularly in high-income countries, the most widely used cut-off point to define a patient as an older adult is 70 years [4]. Older adults represent a very heterogeneous population, with a wide variation regarding functional status, comorbidities, life expectancy and social context. Therefore, treatment decisions can be complex: when chronological age alone is used for decision-making, it can lead to undertreatment or overtreatment, with a negative impact on outcomes such as survival and/or quality of life [5, 6].

Since the mid-1990s, oncologists and geriatricians have tried to integrate geriatric assessment (GA) approaches in the oncology setting; in 2005, the International Society of Geriatric Oncology established recommendations on GA in older patients with cancer, recommending the performance of a GA before oncological treatment as part of the standard of care [6]. GA is defined as a multidimensional health assessment of the older person, an interdisciplinary diagnostic process focusing on determining an older person’s medical, psychosocial and functional capabilities to develop a coordinated and integrated plan for treatment and long-term follow-up [7].

The GA evaluates the most relevant domains for older adults, providing essential information which may help oncologists optimise care. It is recommended for all patients with cancer aged ≥65 years before the start of treatment [8, 9]. GA should be followed by an integrated care plan to address the issues identified by the assessment, with the goal to improve patient outcomes [10]. Information obtained through a GA can improve communication with patients and caregivers and mitigate treatment-associated toxicity [11, 12]. Importantly, the various GA domains require the use of specific tools and follow-up interventions, some of which may not be available everywhere, which may lead some providers to evaluate some domains more often than others [13].

We have previously shown that the use of GA by oncology specialists is low in Mexico. Barriers to its implementation include a lack of qualified personnel, a lack of knowledge on how to perform and interpret a GA, and a perceived lack of time for these assessments [14]. In this study, we aimed to explore factors associated with the evaluation of individual GA domains by Mexican cancer care providers.

## Methods

This is a secondary exploratory analysis of a sequential explanatory mixed-methods study on the use of GA in cancer care in Mexico. The primary study consisted of an online cross-sectional survey (*N* = 196) and follow-up interviews (*n* = 22) administered to Mexican oncology specialist members of the Mexican Society of Oncology. Quantitative and qualitative data collection methods have been previously described [14]. Briefly, members of the Mexican Society of Oncology were invited via email to participate in an online survey that included questions on demographics, awareness of geriatric oncology principles and the use of the GA and other geriatric oncology tools in everyday practice [15]. To select candidates to participate in the interviews, we used maximal variation sampling according to their reported use of GA and characteristics significantly associated with the use of GA in the survey. The interviews contained questions about usual care and physicians’ decision-making process for older adults with cancer, available personnel and infrastructure, reasons for performing/not performing a GA, and barriers and facilitators for performing a GA in their routine practice.

For each GA domain, a separate multivariate logistic regression analysis was performed using the frequency of evaluation of the domain as the dependent variable (dichotomised as never/rarely/sometimes versus most of the time/always). Independent variables included in multivariate analyses were selected based on univariate analysis of factors associated with increased odds of performing a GA among respondents which had a *p* value of <0.1. In addition, self-perceived confidence for treating each GA domain (dichotomised as not at all/a little/mildly confident versus very/completely confident) was included for all models. All statistical analyses were performed using SPSS 21.0 (IBM Corp, Armonk, NY). A *p*-value of <0.05 was considered statistically significant.

For the qualitative component of this exploratory analysis, we focused on interview questions referring to the evaluation of specific GA domains (daily function and cognition). Qualitative data were analysed inductively, based on the survey’s findings using MAXQDA 2020 (VERBI Software, Berlin, Germany).

This study was approved by the institutional review board at Instituto Nacional de Ciencias Médicas y Nutrición Salvador Zubirán (GER-3358-2020-1).

## Results

We obtained 196 valid survey responses (response rate 15.8%): 61.7% of respondents were male, with a median age of 42 years (range 28–86); 50% were surgical oncologists, 30.1% were medical oncologists and 19.4% radiation oncologists. Most oncology specialists (61.7%) reported having a geriatrician available for referrals at their main practice site. Thirty-seven respondents (18.9%) reported performing a multidimensional GA using validated tools when treating older patients with cancer. Other characteristics of the respondents are described in Table 1.

**Figure 1.**
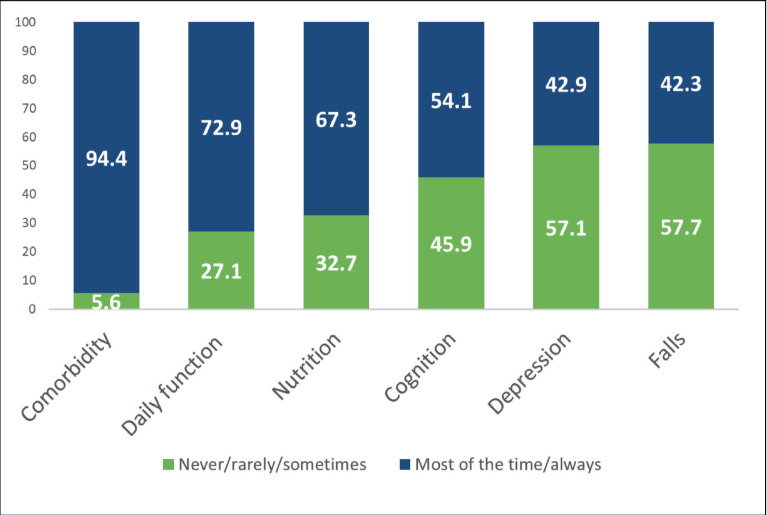
Frequency of evaluation of GA domains by survey respondents (*N* = 196).

The frequency of reported evaluation of each GA domain is shown in Figure 1. Comorbidity was the most frequently assessed domain, with 94.4% of respondents answering that they evaluate it most of the time or always when providing care for older adults with cancer, while falls were the least frequently evaluated domain (42.3%).

**Figure 2.**
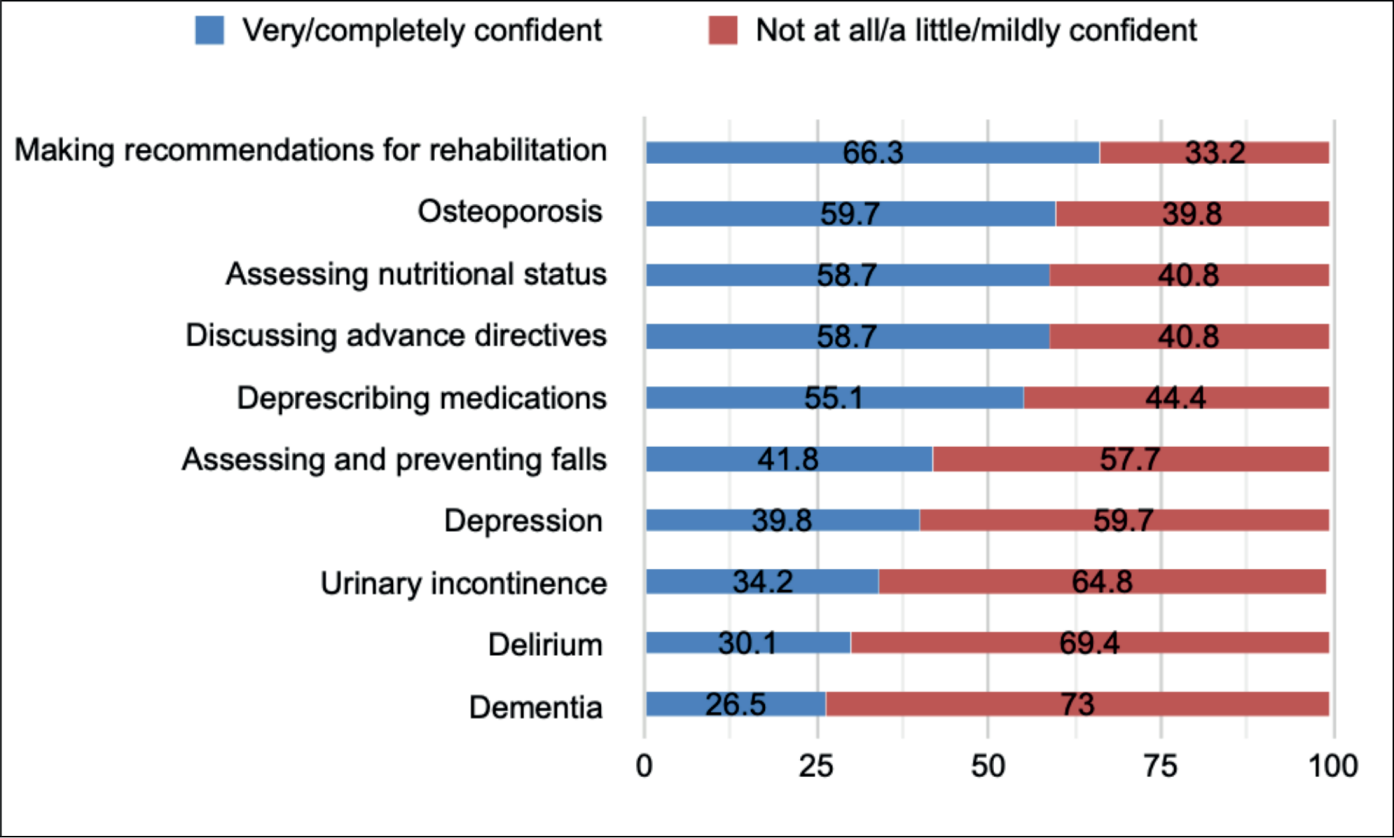
Perceived confidence in managing common situations in older adults.

On univariate analysis, significant variables associated with increased odds of performing a GA included age, gender, medical specialty, practice size of the survey respondent and the presence of a geriatrician in the main practice site. In addition, perceived confidence in managing common situations in older adults relevant for each GA domain (dichotomised as not at all/mildly (reference) versus very/completely) was included in all models (Figure 2). On multivariate analysis, self-perceived confidence in managing dementia (OR 2.72; 95% CI 1.42–5.51, *p* = 0.008) and being a surgical oncologist (OR 2.80; 95% CI 1.29–5.72, *p* = 0.003) were associated with increased odds of evaluating cognition. For nutrition, only self-perceived confidence in nutritional evaluation was associated (OR 3.86; 95% CI 2.0–7.46, *p* < 0.001) with increased odds of conducting a nutritional evaluation. For comorbidities, self-perceived confidence in managing osteoporosis (OR 5.61; 95% CI 1.03–30.4, *p* = 0.046) was associated with increased odds of assessing comorbidities. For falls, significantly associated factors with increased odds of assessment included physician age (OR 1.04; 95% CI 1.01–1.07, *p* = 0.004), larger practice size (OR 0.46; 95% CI 0.23–0.91, *p* = 0.026) and self-perceived confidence in evaluation and prevention of falls (OR 6.31; 95% CI 3.19–12.46, *p* <0.001). Age (OR 1.03; 95% CI 1.01–1.06, *p* = 0.011) and self-perceived confidence in managing depression (OR 2.52; 95% CI 1.33–4.78, *p* = 0.005) were associated with increased odds of evaluating depression. No factors were significantly associated with increased odds of assessing daily function (Table 2).

Of the 22 interview participants, 10 (45.5%) reported performing a multidimensional GA using validated tools when treating older patients with cancer. However, on direct questioning on the exact method of evaluation of one specific domain (cognition), only four participants described using any of the recommended tools included in guidelines such as American Society of Clinical Oncology’s (ASCO’s), i.e., Mini-Cog, the Blessed Orientation-Memory-Concentration test, the Mini-Mental State Examination, or the Montreal Cognitive Assessment. The remaining participants mentioned evaluating cognition through non-validated questions:

- ‘*I ask them questions about mental orientation in space and time*’ (medical oncologist)
- *‘Well, it’s those questions about if they’re conscious and all that; it’s not even if they’re oriented, it’s more on level of consciousness than anything else’* (radiation oncologist)
- ‘*I ask them questions about the past, and they always do really well, better than me, especially on history questions. Some other questions about recent events of the last 2 or 3 months. And other questions regarding their future expectations’* (surgical oncologist)

Some participants reported not having enough confidence in their knowledge of how to perform a cognitive assessment using recommended tools:

- *‘I am aware of the Mini-Mental [State Examination]. I don’t know how to administer it. I am aware of scales for evaluating function in older adults and I don’t know how to use them. So, what I use is the art of medicine. And that’s cool, but the evidence shows that it’s not ideal’* (medical oncologist)
- *‘I’ve done a couple of Mini-Mental [State Examinations], but I don’t feel qualified to administer them, so I prefer that geriatricians do them’* (medical oncologist)

One participant acknowledged that their current method of evaluating cognition was not ideal, and mentioned having insufficient time to use validated tools for this purpose:

- ‘*I ask the patient who is their visit companion, what time it is, what is today’s date, only for orientation. No, it’s just that I don’t have time between consults to evaluate [cognition] using a clock [drawing test] or those…extra things.’* (medical oncologist)

Regarding the assessment of daily function, 12 participants (54%) mentioned using measures such as the Karnofsky Performance Status or the Eastern Cooperative Oncology Group Performance Status scale. Eleven participants (50%) reported assessing function through instrumental activities of daily living (IADLs), activities of daily living (ADLs) or another objective measure of physical performance.

Some participants did not explicitly mention using ADLs or IADLs to assess function but elaborated on evaluating many of these aspects, including advanced ADLs, such as hobbies and working:

- ‘*Since I am not systematic [in performing a GA], sometimes I ask some questions and sometimes other ones, but I ask questions regarding a patient’s self-sufficiency: “Do you live alone? Do you live with someone? Uh, can you do everything on your own? Do you need help with some things? What do you need help with?” And I always focus on…in some way, directly or indirectly, on a patient’s self-sufficiency regarding money since that gives me a general picture of a person’s independence’.* (medical oncologist)

## Discussion

In this exploratory analysis, we found that the reported frequency of evaluation of most specific GA domains by Mexican oncologists was associated with self-perceived confidence in performing that specific evaluation and in implementing strategies for managing the results of the assessment in older adults with cancer. However, when asked about the specific tools used for each evaluation, the respondents acknowledged utilising non-validated tools for conducting assessments.

Older adults with cancer have a higher prevalence of limitations in ADLs and IADLs, as well as a greater number of geriatric syndromes and lower self-assessed health in comparison with older adults without cancer [12]. Identifying these impairments is crucial since this information can serve as a guide for treatment decision-making about a cancer diagnosis in an older adult. Outcomes such as treatment-associated toxicity can be improved in this patient population through focused interventions according to the findings of a GA [12, 16].

When asked about the domains included in their routine assessment of an older adult, the most commonly evaluated domains by surveyed cancer care providers were comorbidities, functional status and nutritional status, whereas depression and cognition were the least evaluated domains, despite the high prevalence of impairments found in older adults with cancer [12]. The majority of surveyed participants referred lower self-perceived confidence in the evaluation and intervention in certain geriatric syndromes, such as dementia, delirium, urinary incontinence, depression and falls. Conversely, participants in our study reported feeling more confident when making recommendations for rehabilitation, treating osteoporosis, evaluating nutritional status and discussing advanced directives. These skills, for which survey respondents reported more confidence, are arguably a part of the recognised competencies of an oncologist, as included in European Society for Medical Oncology-ASCO recommendations for a global curriculum in medical oncology [17]. However, these recommendations also include the development of skills in managing depression and delirium, as well as an appreciation of the different GA domains. A previous report showed that almost half of the community oncologists in the United States felt quite very confident in recognising, evaluating and treating depression, while 39% felt quite very confident in managing delirium and less than 25% in managing dementia [15]. The lower self-perceived confidence in managing depression we found in our study is worrisome, given the prevalence of depression in older patients with cancer [18] and its coexistence with anxiety, pain, impairments in daily function. and other common situations. Delirium is also highly prevalent in patients with advanced cancer [19], and is associated with increased morbidity and mortality, patient and caregiver distress and increased hospitalisation costs. Therefore, it is essential for cancer care providers to be able to prevent, diagnose and treat this condition.

Low-and-middle income countries, including those in Latin America such as Mexico, are affected by a lack of trained personnel and infrastructure for providing care for older adults with cancer [20]. Mexico is among the many countries currently facing a critical shortage of geriatricians: in 2022 there were only 840 board-certified geriatricians in the country [21], for a population of over 130 million, of which 14% are over 60 years old [22]). As for board-certified oncologists, in 2022 there were 1,147 surgical oncologists, 640 medical oncologists, 490 radiation oncologists and 289 gynecologic oncologists (data provided by the Mexican Oncology Board and Mexican Radiotherapy Board). In 2023, a geriatric oncology fellowship programme was launched at Instituto Nacional de Ciencias Médicas y Nutrición Salvador Zubirán in Mexico City, which will train two physicians per year. However, currently, there are no other geriatric oncology training programmes in Mexico or Spanish-speaking Latin America. Also, the majority of oncologists in Mexico never receive dedicated training on the particular needs of older adults with cancer, since geriatric oncology is not a part of medical school or fellowship curricula in the region [23].

With this, a crucial strategy for the implementation of GA in Mexico is the incorporation of geriatric training in the oncology curriculum and vice versa, as well as the development of geriatric oncology continuing professional development activities in geriatric oncology, focusing on the use of screening and assessment tools. This will in turn increase awareness in this area and promote the generation of local experience and research in geriatric oncology.

This is the first study in Latin America and Mexico that reports the frequency of GA domain evaluation by oncology specialists, as well as associated factors. The participation of physicians of multiple specialties in oncology in the survey and interviews is a strength of our study, providing a broad vision of current practices in our country. Among the study limitations is the response rate; however, this response rate is comparable to similar surveys performed in other countries [24]. This also brings a potential response bias with the overrepresentation of those physicians with a special interest in geriatric oncology, which can limit the generalisability of our results. In addition, the proportion of use of GA was self-reported by survey respondents and may not reflect actual adherence to international clinical practice guidelines. We believe the qualitative part of this study was able to elucidate the fact that oncologists’ understanding of what constitutes an evidence-based GA may be limited, and that this highlights the importance of mixed-methods research.

## Conclusion

Self-perceived confidence in evaluating and managing common situations in older adults was associated with the evaluation of GA domains as part of everyday practice among a sample of cancer care providers in Mexico. This analysis supports the use of educational interventions to boost knowledge and confidence regarding the proper use of validated GA tools among oncology specialists.

## Conflicts of interest

Haydee C Verduzco-Aguirre

– Travel, Accommodations, Expenses: AstraZeneca

Héctor Martínez-Said

– Consulting or advisory role: MSD Oncology, Novartis

– Speakers’ bureau: MSD Oncology, Novartis

Gregorio Quintero Beulo

– Employment: Bristol Myers Squibb

– Stock and other ownership interests: Bristol Myers Squibb

– Honoraria: AstraZeneca

– Speakers’ bureau: AstraZeneca

Supriya Gupta Mohile

– Consulting or advisory role: Seattle Genetics

– Research funding: Carevive (Inst)

No other potential conflicts of interest were reported.

## Funding

The authors declare no relevant financial conflicts of interest for this manuscript.

## Author contributions

**Conception and Design:** Haydeé C. Verduzco-Aguirre, Eva Culakova, Supriya G. Mohile, Enrique Soto-Perez-de-Celis.

**Administrative support:** Héctor Martínez- Said, Gregorio Quintero Beulo, Supriya G. Mohile.

**Provision of study materials or patients:** Héctor Martínez-Said, Gregorio Quintero Beulo.

**Collection and assembly of data:** Haydee C. Verduzco-Aguirre, Laura M. Bolaño-Guerra, Javier Monroy Chargoy, Enrique Soto-Perez-de-Celis.

**Data analysis and interpretation:** Haydee C. Verduzco-Aguirre, Laura M. Bolaño Guerra, Eva Culakova, Javier Monroy Chargoy, Supriya G. Mohile, Enrique Soto-Perez-de-Celis.

**Manuscript writing:** All authors.

**Final approval of manuscript:** All authors.

**Accountable for all aspects of the work:** All authors.

## Figures and Tables

**Table 1.** Survey respondent characteristics (*N* = 196).

Characteristics	No (%)
Male	121 (61.7)
Female	72 (36.7)
Missing	3 (1.5)
Age in years, median (range)	42 (28–86)
Medical specialty	
Surgical oncology	84 (42.9)
Medical oncology	59 (30.1)
Radiation oncology	38 (19.4)
Gynecologic oncology	14 (7.1)
Geriatricians available at a main practice site	121 (61.7)
Size of practice	
1–10 patients/day	95 (48.5)
≥11 patients/day	100 (51.0)
Missing	1 (0.5)
Type of practice	
Public and private	75 (38.2)
Public only	67 (34.2)
Private only	52 (26.5)
Missing	2 (1.0)
Main practice at an academic center	82 (41.8)
Performs GA	
Yes	37 (18.9)
No	157 (80.1)
Missing	2 (1.0)

**Table 2.** Multivariate logistic regression analyses of association with evaluation of GA domains.

Domain	Cognition	Nutrition	Comorbidity	Falls	ADLs	Depression
Independent variable	OR (95% CI) *p* value	OR (95% CI) *p* value	OR (95% CI) *p* value	OR (95% CI) *p* value	OR (95% CI) *p* value	OR (95% CI) *p* value
Age	1.01 (0.98–1.04) 0.52	1.00 (0.97–1.03) 0.91	1.03 (0.95–1.11) 0.50	**1.04 (1.01–1.07)** **0.004**	1.01 (0.98–1.04) 0.45	**1.04 (1.01–1.06)** **0.011**
Gender						
Male	Ref	Ref	Ref	Ref	Ref	Ref
Female	0.90 (0.48–1.69) 0.73	1.03 (0.53–2.02) 0.92	3.98 (0.76–21.05) 0.10	0.98 (0.49–1.97) 0.96	1.80 (0.88–3.66) 0.11	0.92 (0.48–1.78) 0.81
Medical specialty						
Medical oncology	Ref	Ref	Ref	Ref	Ref	Ref
Surgical/radiation oncology	**2.80 (1.42–5.51)** **0.003**	1.24 (0.61–2.52) 0.55	4.49 (0.91–22.27) 0.07	0.57 (0.27–1.19) 0.57	0.64 (0.30–1.37) 0.24	0.89 (0.44–1.78) 0.73
Size of practice						
≤10 patients/day	Ref	Ref	Ref	Ref	Ref	Ref
>10 patients/day	0.82 (0.45–1.51) 0.53	0.98 (0.51–1.88) 0.94	1.02 (0.06–1.61) 0.16	**0.47 (0.24–0.91)** **0.026**	0.88 (0.45–1.71) 0.71	0.72 (0.38–1.34) 0.30
Geriatrician available						
No	Ref	Ref	Ref	Ref	Ref	Ref
Yes	0.81 (0.43–1.52) 0.52	0.81 (0.41–1.58) 0.53	1.02 (0.25–4.12) 0.98	0.96 (0.48–1.92) 0.91	1.51 (0.77–2.95) 0.23	1.60 (0.83–3.06) 0.16
Perceived confidence in skill 1	**Dementia**	**Assessing nutrition status**	**Managing polypharmacy**	**Assessing and preventing falls**	**Urinary incontinence**	**Depression**
Not at all-mildly	Ref	Ref	Ref	Ref	Ref	Ref
Very-completely	**2.72 (1.29–5.73)** **0.008**	**3.87 (2.00–7.47)** **<0.001**	1.82 (0.40–8.36) 0.44	**6.31 (3.20–12.46)** **0.001**	1.72 (0.81–3.65) 0.16	**2.52 (1.33-4.78)** **0.005**
Perceived confidence in skill 2			Osteoporosis			
Not at all-mildly	-	-	Ref	-	-	-
Very-completely			**5.61 (1.03–30.50)** **0.046**			
